# Hypoxia-altered cholesterol homeostasis enhances the expression of interferon-stimulated genes upon SARS-CoV-2 infections in monocytes

**DOI:** 10.3389/fimmu.2023.1121864

**Published:** 2023-06-12

**Authors:** Rebekka Bauer, Sofie Patrizia Meyer, Rebecca Raue, Megan A. Palmer, Vanesa Maria Guerrero Ruiz, Giulia Cardamone, Silvia Rösser, Milou Heffels, Fabian Roesmann, Alexander Wilhelm, Dieter Lütjohann, Kathi Zarnack, Dominik Christian Fuhrmann, Marek Widera, Tobias Schmid, Bernhard Brüne

**Affiliations:** ^1^ Institute of Biochemistry I, Faculty of Medicine, Goethe University Frankfurt, Frankfurt, Germany; ^2^ Institute of Medical Virology, University Hospital Frankfurt, Goethe University Frankfurt, Frankfurt, Germany; ^3^ Institute of Clinical Chemistry and Clinical Pharmacology, University of Bonn, Bonn, Germany; ^4^ Buchmann Institute for Molecular Life Sciences (BMLS), Faculty of Biological Sciences, Goethe University Frankfurt, Frankfurt, Germany; ^5^ German Cancer Consortium (DKTK), Partner Site Frankfurt, Frankfurt, Germany; ^6^ Frankfurt Cancer Institute, Goethe University Frankfurt, Frankfurt, Germany; ^7^ Fraunhofer Institute for Translational Medicine and Pharmacology ITMP, Frankfurt, Germany

**Keywords:** hypoxia, immunometabolism, cholesterol, SREBP2, COVID-19, systemic inflammation

## Abstract

Hypoxia contributes to numerous pathophysiological conditions including inflammation-associated diseases. We characterized the impact of hypoxia on the immunometabolic cross-talk between cholesterol and interferon (IFN) responses. Specifically, hypoxia reduced cholesterol biosynthesis flux and provoked a compensatory activation of sterol regulatory element-binding protein 2 (SREBP2) in monocytes. Concomitantly, a broad range of interferon-stimulated genes (ISGs) increased under hypoxia in the absence of an inflammatory stimulus. While changes in cholesterol biosynthesis intermediates and SREBP2 activity did not contribute to hypoxic ISG induction, intracellular cholesterol distribution appeared critical to enhance hypoxic expression of chemokine ISGs. Importantly, hypoxia further boosted chemokine ISG expression in monocytes upon infection with severe acute respiratory syndrome coronavirus type 2 (SARS-CoV-2). Mechanistically, hypoxia sensitized toll-like receptor 4 (TLR4) signaling to activation by SARS-CoV-2 spike protein, which emerged as a major signaling hub to enhance chemokine ISG induction following SARS-CoV-2 infection of hypoxic monocytes. These data depict a hypoxia-regulated immunometabolic mechanism with implications for the development of systemic inflammatory responses in severe cases of coronavirus disease-2019 (COVID-19).

## 1 Introduction

The availability of molecular oxygen (O_2_) is critical for many cellular functions, most notably cellular energy production via oxidative phosphorylation. Thus, various mechanisms evolved to cope with hypoxia, especially with respect to metabolic rewiring in order to protect cells from detrimental effects due to the lack of oxygen (1, 2). Not surprisingly, hypoxia and the resulting adaptive processes are tightly linked to numerous diseases including cancer, as well as metabolic and inflammatory disorders (3). The multilayered crosstalk between metabolic changes and immune responses also forms the basis for the emerging field of immunometabolism (4). While the concept of immunometabolism was termed merely 10 years ago, initial evidence dates back to the early 1990s, when increased expression of the pro-inflammatory cytokine tumor necrosis factor α (TNFα) was observed in rodent models of obesity and was shown to contribute to the development of insulin resistance (5). Along similar lines, altered cholesterol dynamics affect immune functions, as exemplified by observations that upon excessive uptake of low density lipoprotein (LDL)-cholesterol macrophages acquire a pro-inflammatory, foam cell phenotype within atherosclerotic lesions (6). Moreover, using statins to lower plasma LDL-cholesterol concentrations elicited potent anti-inflammatory effects in patients with inflammatory diseases like rheumatoid arthritis or metabolic syndrome (7, 8). Furthermore, intracellular cholesterol trafficking and biosynthetic signaling were shown to activate the inflammasome (9, 10), whereas accumulation of the cholesterol precursor mevalonate induced a trained immunity phenotype in monocyte-derived cells (11). Of note, changes in cholesterol biosynthesis flux also altered anti-viral responses by enhancing interferon (IFN) signaling (12, 13). The connection between IFN signaling and cholesterol metabolism appears to be bidirectional though, as cholesterol biosynthesis enzymes were downregulated in response to viral infection or IFN treatment (14, 15).

In this study, we observed a coinciding transcriptional upregulation of cholesterol biosynthesis enzymes and IFN-stimulated genes (ISGs) in hypoxic monocytes. Mechanistically, hypoxia-evoked changes in cholesterol dynamics enhanced toll-like receptor (TLR) signaling and consequently IFN responses. Hypoxia further increased chemokine ISG production in monocytes upon infection with severe acute respiratory syndrome coronavirus type 2 (SARS-CoV-2), thereby providing a novel concept how hypoxemia, i.e., low blood oxygen levels, might favor systemic inflammation in severe cases of coronavirus disease-2019 (COVID-19).

## 2 Materials and methods

### 2.1 Chemicals

All chemicals were purchased from Thermo Fisher Scientific GmbH (Dreieich, Germany), if not indicated otherwise. Fatostatin hydrobromide, TAK-242, IKK-16 hydrochloride, lathosterol, 7-dehydrocholesterol, and desmosterol were purchased from Cayman Chemical (Ann Harbor, MI, USA), PF-429242 dihydrochloride, ketoconazole, methyl-β-cyclodextrin-complexed (water-soluble) cholesterol, mevalonolactone, and geranylgeraniol from Sigma-Aldrich (Taufkirchen, Germany), U18666A from Enzo Life Sciences (Lausen, Switzerland), simvastatin from Selleck Chemicals (Planegg, Germany), NB-598 maleate from Adooq Bioscience (Irvine, CA, USA), enpatoran from TargetMol (Wellesley Hills, MA, USA), T0901317 from Tocris (Wiesbaden-Nordenstadt, Germany), TJ-M2010-5 from Hycultec GmbH (Beutelsbach, Germany), and BX-795 from MedChemExpress (Monmouth Junction, NJ, USA).

### 2.2 Cell culture

THP-1 cells were obtained from ATCC, and THP-1 STING- and MAVS-KO cells as well as the corresponding original WT THP-1 cells were kindly provided by Prof. Veit Hornung (LMU Munich, Germany) (16). THP-1 cells were cultured in Roswell Park Memorial Institute (RPMI) 1640 medium, supplemented with 100 U/mL penicillin, 100 µg/mL streptomycin, and 10% or 5% FBS (Capricorn Scientific GmbH, Ebsdorfergrund, Germany or Sigma-Aldrich), dependent on the cholesterol concentration of the respective FBS batch. For experiments performed under low FBS levels, the percentage of FBS was reduced to 1/10^th^ of the FBS amount used for maintaining the cells. Cells were kept at 37°C in a humidified atmosphere with 5% CO_2_. For hypoxic incubations, cells were transferred to a hypoxia workstation (SCI-tive, Baker Ruskinn, Bridgend, South Wales, UK) at 37°C with 5% CO_2_ and 1% O_2_.

### 2.3 IFNAR neutralization

THP-1 cells were treated with 5 µg/mL α-IFNAR2 antibody (Clone MMHAR-2 Mab, PBL assay science, Piscataway, NJ, USA; cat. no. 21385) or IgG2a isotype control antibody (Clone C1.18.4, Bio X Cell, Lebanon, NH, USA; cat. no. BE0085) prior to normoxic or hypoxic incubation for 24 h.

### 2.4 SARS-CoV-2 infection

Lung-derived A549-AT cells, constitutively expressing ACE2 and TMPRSS2 (17), were infected with SARS-CoV-2 strain FFM1 (accession number MT358638.1) (18) using a multiplicity of infection (MOI) of 0.1 in Minimum Essential Medium (MEM) containing 1% FBS, 100 U/mL penicillin, 100 µg/mL streptomycin, and 4 mM L-glutamine (all Sigma-Aldrich). After 1 h inoculation at 37°C and 5% CO_2_, cells were washed once with PBS and fresh medium was added. 48 h post infection (hpi), virus-containing supernatants were centrifuged and stored at -80°C until further usage.

Monocytic THP-1 cells were cultured for 24 h in RPMI 1640 with 1% FBS, 100 U/mL penicillin, and 100 µg/mL streptomycin (all Sigma-Aldrich) at 37°C with 5% CO_2_ and either 21% or 1% O_2_. Optionally, 10 µM fatostatin, 10 µM TAK-242, or 0.1% DMSO (Carl Roth, Karlsruhe, Germany) were added 1 h before starting hypoxic cultures. Experiments involving SARS-CoV-2 were carried out in an oxygen-adjustable incubator in a biosafety level 3 (BSL3) facility. After 24 h normoxic or hypoxic incubations, THP-1 cells were infected with the SARS-CoV-2 containing virus supernatants. Supernatants of non-infected A549-AT were used as controls. Cells were washed 1 hpi with PBS, and either directly lysed for RNA isolation, or incubated for additional 5 h, before freezing debris-free supernatants at -80°C for subsequent ELISAs and lysing cells for RNA isolation. Sample inactivation for further processing was performed with previously evaluated methods (19).

### 2.5 Stimulation with SARS-CoV-2 spike protein

THP-1 cells were pre-incubated for 24 h under normoxia or hypoxia before 5 µg/mL recombinant SARS-CoV-2 spike trimer (S1+S2) (BPS Bioscience, San Diego, CA, USA; cat. no. 100728) or 0.04% glycerol (Sigma-Aldrich) as vehicle control were added for additional 8 h normoxic or hypoxic incubations.

### 2.6 RNA isolation, reverse transcription, and quantitative polymerase chain reaction

Total RNA from THP-1 cells was extracted using either TRIzol or the RNeasy mini kit (for SARS-CoV-2 experiments; Qiagen, Hilden, Germany) according to the manufacturer’s instructions. The Maxima First Strand cDNA synthesis kit was used for reverse transcription and qPCR analyses were performed using PowerUp SYBR Green Master Mix on QuantStudio 3 and 5 PCR Real-Time Systems (Thermo Fisher Scientific). Primers were ordered from Biomers (Ulm, Germany) and are listed in **Supplementary Table S1**, except the primer for *IRF7* (Hs_IRF7_1_SG QuantiTect Primer Assay), which was purchased from Qiagen.

### 2.7 Differential gene expression analysis

Previously, we characterized transcriptome-wide changes in *de novo* synthesis and RNA stability under hypoxia in monocytes by a metabolic labeling approach. Here, we focused on total mRNA changes within the previously published comprehensive metabolic RNA sequencing data of THP-1 cells incubated for 8 h and for 72 h under hypoxia (acute hypoxia (= AH) and chronic hypoxia (= CH), respectively), or under normoxia (N) (GSM5994456 to GSM5994464) (20). For differential gene expression analyses, raw reads were quality-, adapter-, and polyA-trimmed using Cutadapt (21) and unique molecular identifier and linker sequences were removed before the processed reads were aligned to the human genome (GRCh38) with Ensembl gene annotation (release 80) using STAR (version 2.7.6a) (22). Transcript counts were determined using htseq-count with default parameters (23) and Ensembl gene annotation (release 80). Differentially expressed genes were determined using DESeq2 in R (24). Log_2_-transformed fold changes in genes were shrunken using the estimator “ashr”. Adjusted *p*-values (*padj*) were determined using Benjamini-Hochberg correction, and differentially regulated transcripts between N, AH, and CH were visualized with ComplexHeatmaps (25). Hereto, read counts were corrected for library size using DESeq2 size factors and subjected to a row-wise *z*-score normalization. Transcripts were grouped into three groups by *k*-means clustering. For the identification of enriched functional annotation clusters, transcripts downregulated (first cluster) or upregulated (second and third clusters) by hypoxia were analyzed separately using the Database for Annotation, Visualization and Integrated Discovery (DAVID) against the gene sets “GOTERM_BP_DIRECT” and “UP_KW_BIOLOGICAL_PROCESS” (26, 27). A list of all detected transcripts (basemean > 0, for all conditions) served as background data set.

### 2.8 Interferome analysis

Transcripts constituting the functional annotation cluster “immune cell activation” within the hypoxic upregulated transcripts were used as input for Interferome v2.01 (28). Interferome v2.01 compared the input transcripts with a comprehensive database of collected gene expression data from different cell types after treatment with type I, II, or III IFNs. For further analyses, we used only the interferon-stimulated genes (ISGs) from all identified interferon-regulated genes (IRGs) within the “immune cell activation” cluster. The distribution of the putative type I, II, and/or III IFN targets was visualized using VennDiagram (29), and the library-size and row-wise *z*-score normalized read counts of the so-identified ISGs under N, AH, and CH were visualized with ComplexHeatmaps (25).

### 2.9 Immunoblots

All reagents used for immunoblotting were purchased from Sigma-Aldrich, if not indicated otherwise. THP-1 cells were resuspended in lysis buffer (10 mM Tris-HCl, 6.65 M Urea, 10% glycerol, 1% SDS (Carl Roth), pH 7.4; freshly supplemented with 1 mM DTT (Carl Roth), protease inhibitor and phosphatase inhibitor mixes (cOmplete and phosSTOP, respectively (Roche, Grenzach-Wyhlen, Germany)), and sonicated. 70 µg total protein were separated by sodium dodecylsulfate polyacrylamide gel electrophoresis and transferred onto nitrocellulose membranes (GE Healthcare, Chalfont St Giles, UK). Proteins were detected using specific antibodies for LSS (Proteintech, Planegg-Martinsried, Germany; cat. no. 13715-1-AP), β-tubulin (Abcam, Cambridge, UK; cat. no. ab7780), pSTAT1 (Tyr701; Cell Signaling, Leiden, Netherlands; cat. no. 7649S), or STAT1 (Cell Signaling; cat. no. 9172S) and appropriate IRDye secondary antibodies (LI-COR Biosciences, Bad Homburg, Germany), and visualized using the Odyssey infrared imaging system (LI-COR Biosciences).

### 2.10 Immunofluorescent staining

THP-1 cells were incubated for 8 h under normoxia or hypoxia and subsequently fixed with ROTI^®^Histofix (Carl Roth) for 10 min at 4°C. After transferring to object slides using a cytospin centrifuge, cells were permeabilized with 0.1% triton X in PBS for 10 min, followed by blocking with 10% normal goat serum (Sigma-Aldrich) with 100 mM glycine. Primary rabbit anti-SREBP2 antibody (Cayman Chemical; cat. no. 10007663) was incubated at 1:500 in 2% normal goat serum overnight at 4°C. F(ab’)2 goat anti-rabbit IgG Alexa fluor™ plus 488 secondary antibody (Thermo Fisher Scientific; cat. no. A48282) was incubated at 1:500 in 2% normal goat serum for 45 min at room temperature. Cells were counterstained with 1 µg/mL 4’,6-diamidino-2-phenylindole (DAPI) for 1 min. Whole slide scans were performed using Vectra Polaris (Akoya Biosciences, Marlborough, MA, USA) at 20x magnification. Image analysis was performed in QuPath v0.4.2 (30), cell detection with a 5 μm expansion was performed on annotations of the whole cytospin area. Mean nuclear intensity values per cell were generated for analysis.

### 2.11 ELISAs

CCL2 and CXCL10 protein levels in the supernatants of SARS-CoV-2 infected THP-1 cells were quantified using SimpleStep ELISA kits from Abcam according to the manufacturer’s instructions.

### 2.12 Sterol measurements

THP-1 cells were incubated for up to 24 h under normoxia or hypoxia. Optionally, cells were pre-incubated with 1 µM simvastatin, 10 µM NB-598, 10 µM ketoconazole, or 0.1% DMSO for 1 h. Sterol content was determined by gas chromatography-mass spectrometry-selected ion monitoring (GC-MS-SIM) as previously described (31–33). Briefly, cell pellets were dried in a speedvac concentrator (12 mbar; Savant AES 1000) and weighed. Cholesterol and cholesterol precursors were extracted using chloroform. After alkaline hydrolysis, the concentrations of the cholesterol precursors lanosterol, 24,25-dihydrolanosterol, lathosterol, and desmosterol were measured with GC-MS-SIM in selected ion monitoring mode. The trimethylsilyl-ethers of the sterols were separated on a DB-XLB (30 m length x 0.25 mm internal diameter, 0.25 µm film) column (Agilent Technologies, Waldbronn, Germany) using the 6890N Network GC system (Agilent Technologies). Epicoprostanol (Steraloids, Newport, RI, USA) was used as an internal standard, to quantify the non-cholesterol sterols (Medical Isotopes, Pelham, NH, USA) on a 5973 Network MSD (Agilent Technologies). Total cholesterol was measured by GC-flame ionization detection on an HP 6890 GC system (Hewlett Packard, Waldbronn, Germany), equipped with a DB-XLB (30 m length x 0.25 mm internal diameter, 0.25 µm film) column (Agilent Technologies) using 5α-cholestane (Steraloids) as internal standard.

### 2.13 Statistical analysis

Data are reported as means ± SEM of at least three independent experiments. Statistical analyses were carried out using GraphPad Prism v9.3.1 (GraphPad Software, San Diego, CA, USA) or R v4.0.5 (34). Statistical significance was estimated either using two-tailed paired t-test, one-way or two-way repeated measures ANOVA with Holm-Šídák’s multiple comparisons test as applicable. If residuals were assumed to be not normally distributed (based on quantile-quantile (Q-Q) plots), data were log-transformed before statistical testing.

## 3 Results

### 3.1 Hypoxia enhances expression of cholesterol biosynthesis enzymes and increases IFN signaling

**Figure 1:**
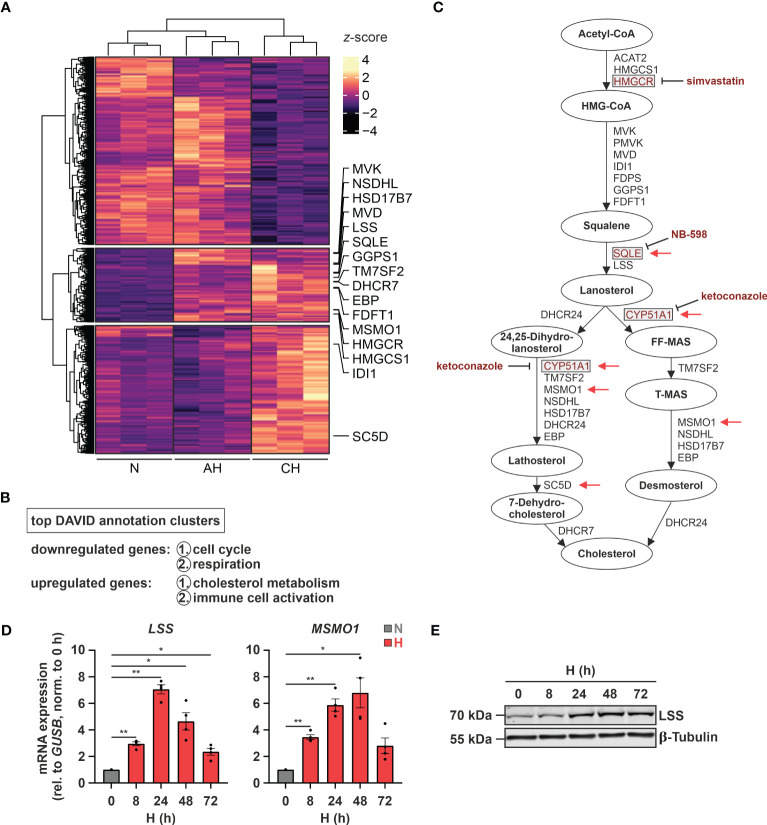
Hypoxia induces cholesterol biosynthesis enzymes. **(A)** RNA expression in THP-1 cells exposed to hypoxia (H; 1% O_2_) for 8 h (acute hypoxia = AH) or 72 h (chronic hypoxia = CH) was determined relative to normoxic THP-1 cells (N; 21% O_2_) by RNA-Seq (n = 3). Differentially expressed genes (*padj* < 0.05, |log2FC| > 0.3) were visualized in a heatmap (*z*-score normalized counts) and categorized by *k*-means clustering. Cholesterol biosynthesis genes are highlighted. **(B)** Top two functional annotation clusters of down- or upregulated transcripts identified by DAVID (26, 27). **(C)** Schematic representation of the cholesterol biosynthesis cascade. Oxygen-demanding steps are highlighted by red arrows and selected inhibitors as well as their target enzymes are marked in red. **(D, E)** THP-1 cells were incubated under N (grey) or H (red) for the indicated times (n = 4). **(D)** *LSS* and *MSMO1* mRNA expression was analyzed by RT-qPCR and normalized to *GUSB* expression. **(E)** LSS protein expression was determined by Western blot analysis. β-tubulin served as loading control. The blot is representative of four independent experiments. All data are means ± SEM and were statistically analyzed using one-way repeated measures ANOVA with Holm-Šídák’s multiple comparisons test (*p < 0.05, **p < 0.01).

Since hypoxia is a major contributing factor to various immune system-associated diseases, we determined RNA dynamics in human monocytic THP-1 cells in response to acute (8 h 1% O_2_ = AH) and chronic (72 h 1% O_2_ = CH) hypoxia (20). In line with the major regulatory impact of hypoxia, 2632 transcripts appeared differentially expressed (*padj* < 0.05, |log2FC| > 0.3) between normoxia (21% O_2_ = N) and hypoxia (AH and/or CH), however, following different regulatory dynamics (**Figure 1A**). While 1268 targets decreased under acute and/or chronic hypoxia (first cluster), 1364 targets increased either cumulatively during hypoxic incubations (second cluster) or only in response to CH (third cluster) (**Supplementary Table S2**). Functionally, cell cycle and respiration emerged as top enriched annotations amongst the downregulated transcripts, whereas cholesterol metabolism and immune cell activation were enriched within the upregulated candidates (**Figure 1B**; **Supplementary Table S2**). In fact, the majority of enzymes involved in the cholesterol biosynthesis cascade were upregulated, mostly already under AH (**Figures 1A, C**). To obtain further insights into the dynamics of cholesterol biosynthesis gene expression under hypoxia, we determined expression of representative genes in THP-1 cells over a time course of up to 72 h of hypoxia. mRNA expression of the selected candidates *lanosterol synthase* (*LSS*) and *methylsterol monooxygenase 1* (*MSMO1*) increased after 8 h of hypoxia, reaching maximal levels at 24 - 48 h, thereafter decreasing (**Figure 1D**). In line, LSS protein expression increased after 24 h and remained elevated up to 72 h of hypoxia (**Figure 1E**).

**Figure 2:**
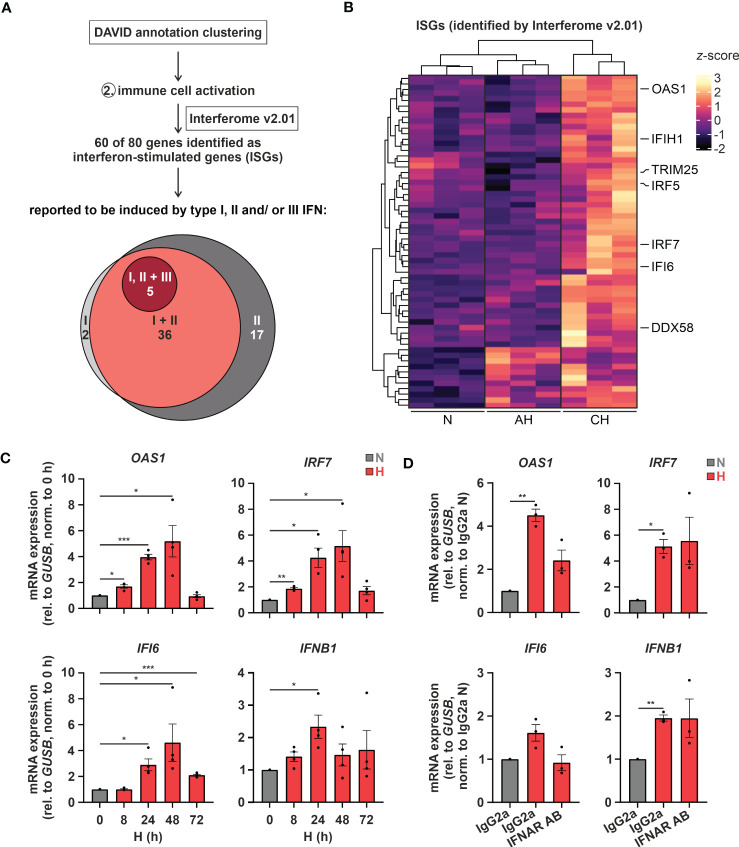
Hypoxia increases interferon (IFN) signaling. **(A)** IFN-stimulated genes (ISGs) within the transcripts constituting the functional annotation cluster “immune cell activation” were identified using Interferome v2.01 (28). Venn diagram depicts proposed ISGs regulated by type I, II, and/or III IFNs according to Interferome v2.01. **(B)** Heatmap representing differentially expressed, hypoxia-induced ISGs (*z*-score normalized count data) under normoxia (N), acute hypoxia (AH; 8 h), and chronic hypoxia (CH; 72h) (n = 3; *padj* < 0.05, |log2FC| > 0.3). Selected ISGs are highlighted. **(C)** THP-1 cells were incubated under N (grey) or H (red) for the indicated times (n = 4) or **(D)** treated with 5 µg/mL α-IFNAR2 antibody or an IgG2a-isotype control and incubated under N (grey) or H (red) for 24 h (n = 3). *OAS1, IRF7, IFI6*, and *IFNB1* mRNA expression was analyzed by RT-qPCR and normalized to *GUSB* expression. All data are means ± SEM and were statistically analyzed by one-way repeated measures ANOVA with Holm-Šídák’s multiple comparisons test (*p < 0.05, **p < 0.01, ***p < 0.001).

Of note, changes in cholesterol metabolism were previously linked to altered immune responses (35), especially to interferon (IFN) signaling (12, 13). Since “immune cell activation” emerged as the second most enriched function within the differentially induced genes in hypoxic THP-1 monocytes (**Figure 1B**), we determined the contribution of interferon-stimulated genes (ISGs) to the hypoxia-induced immune response using the Interferome v2.01 database (28). Of note, 75% (60 of 80) of the immune activation-associated transcripts regulated under hypoxia in THP-1 cells were potential ISGs. Of these the vast majority, i.e., 60% (= 36), were proposed targets of both type I and II IFNs, 28% (= 17) were exclusive type II IFN targets, two exclusive type I IFN targets, and five associated with type I, II, as well as III IFNs (**Figure 2A**; **Supplementary Table S3**). Interestingly, in contrast to cholesterol biosynthesis-associated targets most ISGs (49 of 60) were predominantly induced under CH (**Figure 2B**; **Supplementary Table S3**). Refined hypoxia time course experiments validated maximal induction of *2’-5’-oligoadenylate synthetase 1* (*OAS1*), *interferon regulatory factor 7* (*IRF7*), and *interferon α-inducible protein 6* (*IFI6*) at 48 h of hypoxia, whereas *interferon β1* (*IFNB1*) was maximal after 24 h (**Figure 2C**). To determine whether early IFN-β induction in hypoxia might contribute to the expression of some of the ISGs increasing later on, we blocked IFN-β-receptor-dependent signaling in hypoxic THP-1 cells (24 h) using an α-interferon-α/β-receptor subunit 2 (IFNAR2) antibody (5 µg/mL). While *IFNB1* and *IRF7* expression was not influenced by IFNAR2 neutralization compared to the respective IgG2a-isotype control, *OAS1* and *IFI6* induction were markedly reduced upon IFNAR2 blockage (**Figure 2D**). In line with activation of type I IFN receptor signaling, the downstream effector signal transducer and activator of transcription 1 (STAT1) was phosphorylated (Tyr701) after 8 - 24h of hypoxia (**Supplementary Figure 1**).

Taken together, hypoxia enhances the expression of nearly all enzymes of the cholesterol biosynthesis cascade and at the same time induces a broad range of ISGs in monocytic THP-1 cells, in part by a secondary IFNAR-dependent amplification loop.

### 3.2 Hypoxic ISG induction is not directly affected by cholesterol biosynthesis intermediates

**Figure 3:**
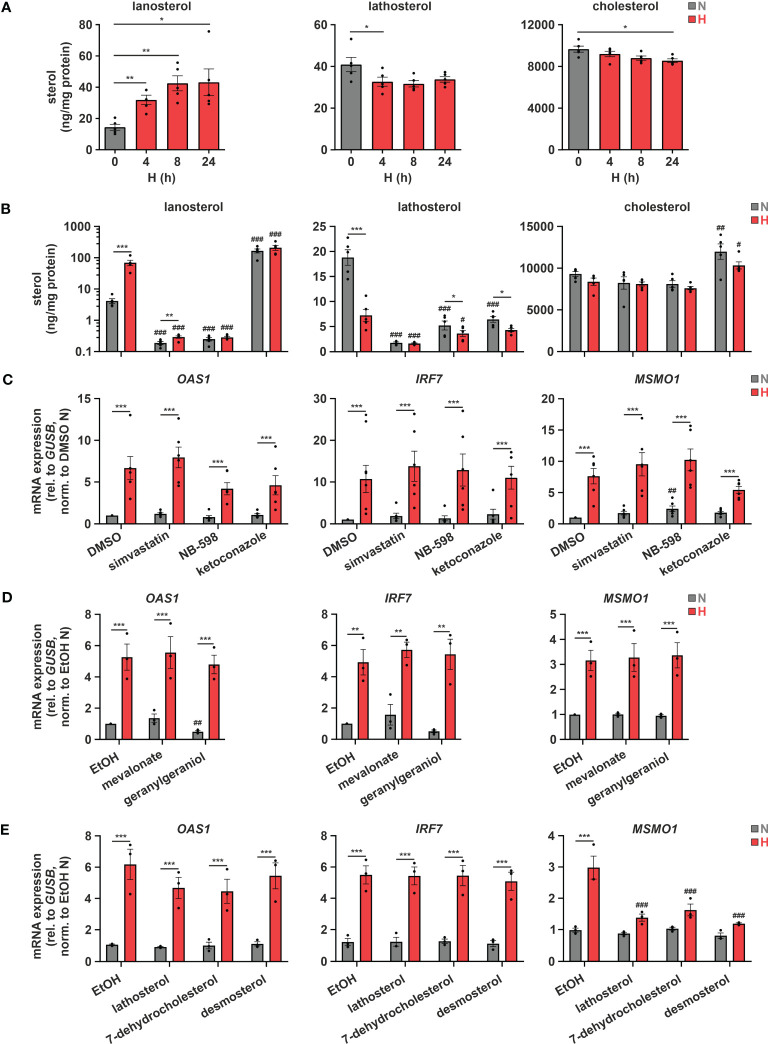
Hypoxic ISG induction is not directly affected by cholesterol biosynthesis intermediates. **(A)** THP-1 cells were incubated under N (grey) or H (red) for the indicated times (n = 5). Sterol levels were measured by GC-MS. **(B, C)** THP-1 cells were pre-incubated for 1 h with 1 µM simvastatin, 10 µM NB-598, 10 µM ketoconazole, or DMSO, prior to incubation under N (grey) or H (red) for 24 h (n = 5). **(B)** Cellular sterol levels were measured by GC-MS. **(C)** *OAS1*, *IRF7*, and *MSMO1* mRNA expression was analyzed by RT-qPCR and normalized to *GUSB* expression. **(D, E)** THP-1 cells were pre-incubated for 1 h with **(D)** the early cholesterol precursors mevalonate (300 µM) or geranylgeraniol (15 µM) (n = 3), **(E)** the late cholesterol precursors lathosterol, 7-dehydrocholesterol, or desmosterol (each 5 µM) (n = 3), or appropriate solvent controls, prior to incubation under N (grey) or H (red) for 24 h. *OAS1*, *IRF7*, and *MSMO1* mRNA expression was analyzed by RT-qPCR and normalized to *GUSB* expression. All data are means ± SEM and were statistically analyzed using one-way repeated measures ANOVA with Holm-Šídák’s multiple comparisons test **(A)**, or two-way repeated measures ANOVA with Holm-Šídák’s multiple comparisons test **(B–E)** (*p < 0.05, **p < 0.01, ***p < 0.001; #*p* < 0.05, ##*p* < 0.01, ###*p* < 0.001 (compared to respective solvent controls)).

Considering previous reports showing that a disturbance in cholesterol metabolism may increase IFN signaling (12, 13), we asked whether changes in cholesterol metabolism might also contribute to ISG induction under hypoxia. To this end, we initially measured sterol levels in THP-1 cells in the course of hypoxia. In accordance with several oxygen-demanding steps within the cholesterol biosynthesis cascade (**Figure 1C**, red arrows), lathosterol and desmosterol, i.e., sterol intermediates downstream of the major oxygen-demanding reactions, were reduced, while the early cholesterol precursors lanosterol and 24,25-dihydrolanosterol markedly accumulated under hypoxia (**Figure 3A**; **Supplementary Figure 2**). Levels of total cholesterol appeared to be only minimally attenuated by reduced oxygen, though at much higher total amounts than the other sterols. Noteworthy, while changes in lanosterol and lathosterol were almost maximal already at 4 h of hypoxia (**Figure 3A**), expression of cholesterol biosynthesis enzymes as well as of ISGs remained unaltered at this early time point (**Supplementary Figure 3**), suggesting that changes in cholesterol metabolites might contribute to the observed gene expression changes. To prevent or mimic hypoxic accumulation of lanosterol and 24,25-dihydrolanosterol, we next pre-treated THP-1 cells with either the 3-hydroxy-3-methylglutaryl-CoA reductase (HMGCR) inhibitor simvastatin (1 µM), the squalene epoxidase (SQLE) inhibitor NB-598 (10 µM), or the cytochrome P450 51A1 (CYP51A1, lanosterol 14α-demethylase) inhibitor ketoconazole (10 µM) for 1 h before incubating them for 24 h under normoxia or hypoxia (see **Figure 1C** for interventions). Inhibition of HMGCR and SQLE significantly reduced lanosterol and lathosterol levels already under normoxia and prevented hypoxia-mediated accumulation of lanosterol (**Figure 3B**). As expected, inhibition of the lanosterol/24,25-dihydrolanosterol-metabolizing enzyme CYP51A1 reduced the late intermediate lathosterol, while it massively increased lanosterol under normoxia, even overruling the hypoxia-induced increase. As observed under hypoxia, cholesterol levels displayed only slight changes in response HMGCR and SQLE inhibition, but surprisingly increase markedly upon ketoconazole treatment. Despite pronounced changes in sterol *de novo* synthesis, all three inhibitors only minimally affected *MSMO1* levels under normoxia and did not alter its hypoxic induction (**Figure 3C**). These findings suggest that total cholesterol levels are an imprecise measure to predict changes in intracellular cholesterol dynamics. Similarly, *OAS1* and *IRF7*, i.e., IFNAR-dependent and -independent ISGs, respectively, remained unaffected by the three inhibitors under normoxia and hypoxia, indicating that accumulation of early cholesterol precursors did not contribute to hypoxic ISG induction. To determine if cholesterol biosynthesis intermediates might still be involved in hypoxic ISG induction, we supplemented THP-1 cells with early (mevalonate (300 µM), geranylgeraniol (15 µM)) or late cholesterol precursors (lathosterol, 7-dehydrocholesterol, desmosterol (5 µM each)) prior to 24 h of hypoxia. Corroborating the observation that cholesterol biosynthesis inhibitors did not alter hypoxic ISG induction, supplementation of neither early nor late cholesterol precursors substantially attenuated the hypoxia-mediated increase in *OAS1* and *IRF7* expression (**Figures 3D, E**). In line with the oxygen requirements for cholesterol biosynthesis, early cholesterol intermediates did not affect hypoxic *MSMO1* induction (**Figure 3D**), while late intermediates almost completely prevented the hypoxic increase in *MSMO1* expression (**Figure 3E**).

**Figure 4:**
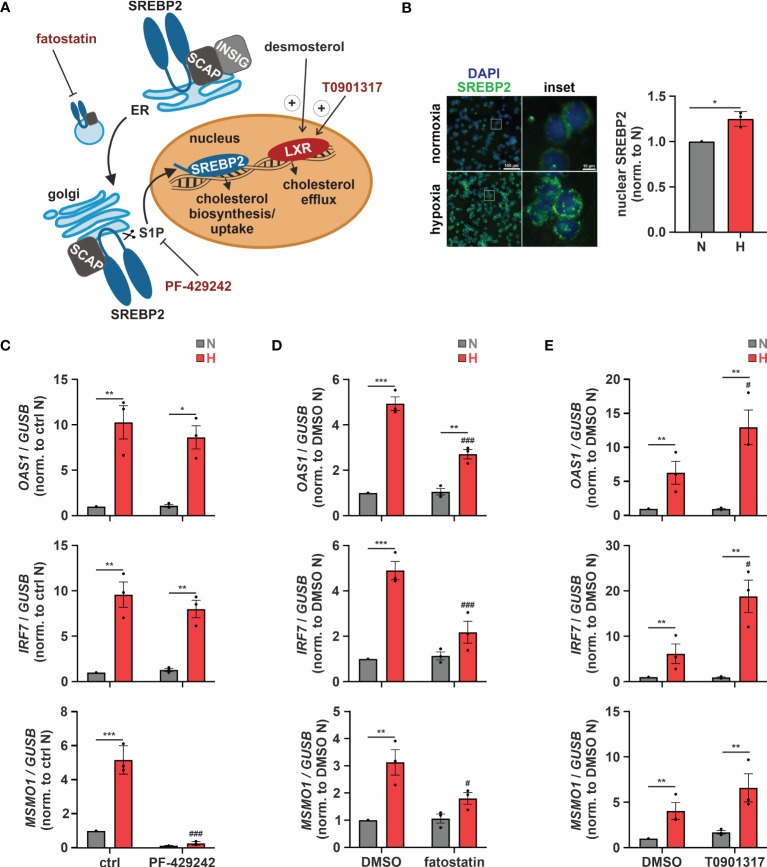
Altered cholesterol homeostasis affects hypoxic ISG induction. **(A)** Overview of the mechanisms of activation of the cholesterol-associated transcription factors LXR and SREBP2. The LXR agonist T0901317 as well as the SREBP2 inhibitors PF-429242 and fatostatin are highlighted in red. **(B)** THP-1 cells were incubated under N (grey) or H (red) for 8 h (n = 3). Immunofluorescence staining for SREBP2 (green) was performed. Nuclei were counterstained with DAPI (blue). Mean fluorescence intensity was quantified for nuclear SREBP2. Images are representative of three independent experiments. **(C–E)** THP-1 cells were pre-incubated for 1 h with **(C)** 1 µM PF-429242, **(D)** 10 µM fatostatin, **(E)** 1 µM T0901317, or appropriate solvent controls, prior to incubation under N (grey) or H (red) for 24 h (n = 3). *OAS1*, *IRF7*, and *MSMO1* mRNA expression was analyzed by RT-qPCR and normalized to *GUSB* expression. Data are means ± SEM and were statistically analyzed using two-tailed paired t-test **(B)**, or two-way repeated measures ANOVA with Holm-Šídák’s multiple comparisons test **(C–E)** (*p < 0.05, **p < 0.01, ***p < 0.001; #*p* < 0.05, ###*p* < 0.001 (compared to ctrl or DMSO, respectively)).

Subcellular changes in cholesterol concentrations provide a rheostat to control the activities of the transcription factors sterol regulatory element-binding protein 2 (SREBP2), which is activated after sterol depletion at the endoplasmic reticulum (ER) to enhance cholesterol biosynthesis and uptake (36), and liver X receptor (LXR), which is activated by desmosterol or oxysterols to reduce cholesterol uptake and enhance cholesterol export (**Figure 4A**) (37). First, we addressed the involvement of SREBP2, the master regulator of the enzymes involved in cholesterol biosynthesis, in the hypoxic induction of the cholesterol biosynthesis enzymes. Indeed, after 8 h of hypoxia nuclear SREBP2 levels, reflecting active SREBP2, were increased (**Figure 4B**). Furthermore, pre-incubation of THP-1 cells with the established SREBP2 inhibitors PF-429242 (1 µM) or fatostatin (10 µM) effectively blocked hypoxic *MSMO1* induction (**Figures 4C, D**). While SREBP2 was previously described to directly bind and activate IFN response genes (38), and inhibition of SREBP2 cleavage and release from the Golgi with the S1P (site 1 protease) inhibitor PF-429242 completely blocked SREBP2 target expression even under normoxia, it did not affect hypoxic *OAS1* and *IRF7* induction (**Figure 4C**). In contrast, inhibition of SREBP2 activation with fatostatin (10 µM), which selectively blocked the hypoxic increase in *MSMO1* expression, also attenuated the hypoxic ISG expression (**Figure 4D**). To further test if cholesterol homeostasis changes might affect ISG expression, we incubated THP-1 cells with the LXR agonist T0901317 (1 µM). In line with reduced cholesterol biosynthesis and desmosterol levels, expression of the cholesterol exporter *ATP binding cassette subfamily A member 1* (*ABCA1*), a proto-typical LXR target, was reduced under hypoxia (**Supplementary Figure 4**). Interestingly, small molecule-based activation of LXR significantly increased the hypoxic ISG induction (**Figure 4E**).

Conclusively, our data indicate that while the hypoxic ISG induction in monocytes is not directly affected by changes in cholesterol biosynthetic flux, modulation of subcellular cholesterol dynamics might contribute to the enhanced ISG expression under hypoxia.

### 3.3 Intracellular cholesterol distribution determines hypoxic chemokine ISG induction

**Figure 5:**
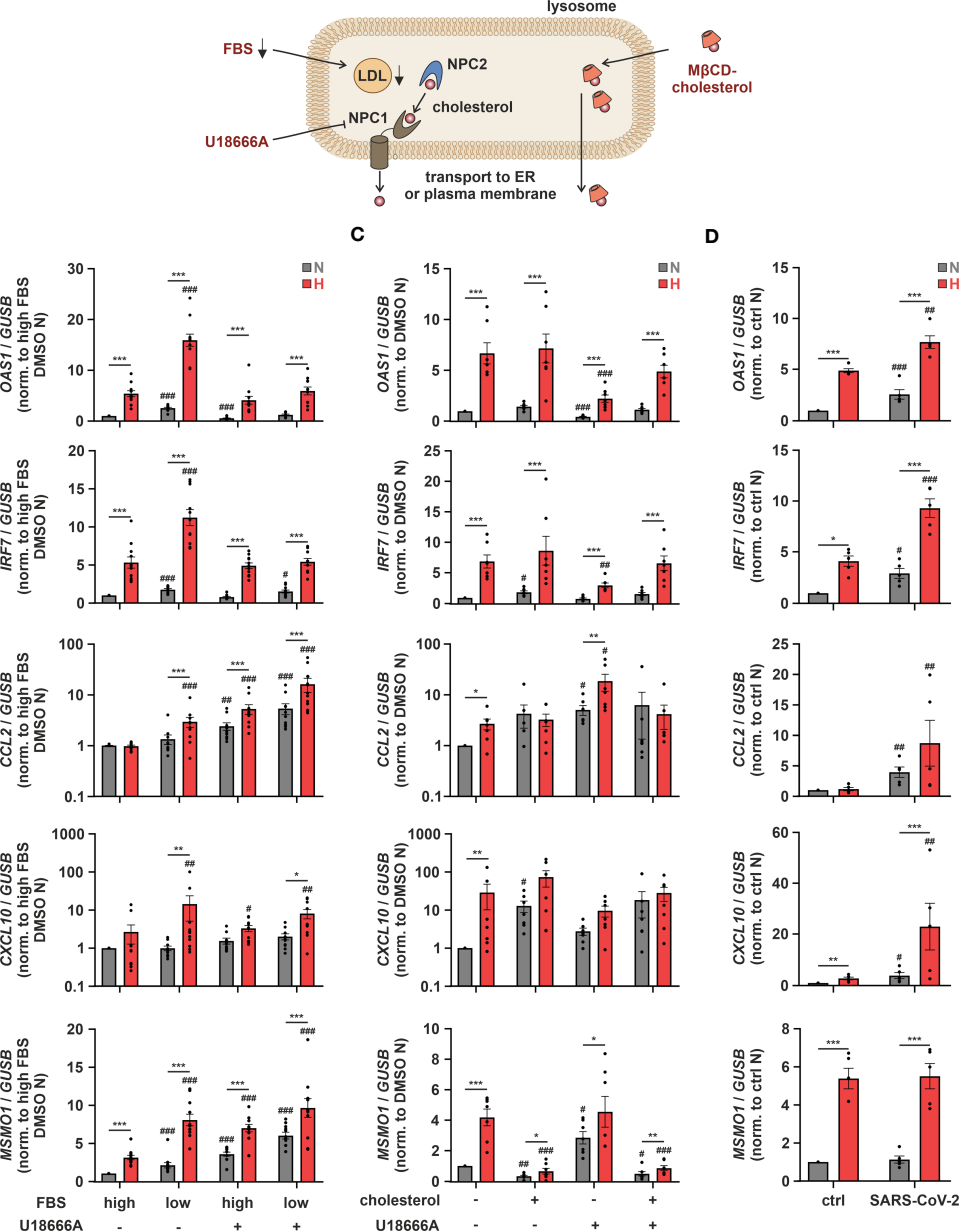
Intracellular cholesterol distribution determines hypoxic chemokine ISG induction. **(A)** Overview of the lysosomal cholesterol distribution machinery. Relevant interventions are indicated in red. **(B)** THP-1 cells were pre-incubated for 1 h with 5 µM U18666A or DMSO in medium containing high or low levels of FBS prior to incubation under N (grey) or H (red) for 24 h (n = 11). **(C)** THP-1 cells were pre-incubated for 1 h with 5 µM U18666A or DMSO in medium containing low levels of FBS ± 0.25 mg/mL methyl-β-cyclodextrin-complexed cholesterol prior to incubation under N (grey) or H (red) for 24 h (n = 7). **(D)** THP-1 cells were incubated under N (grey) or H (red) for 24 h in medium containing low levels of FBS prior to infection with SARS-CoV-2 (strain FFM1) under N. RNA was isolated 1 hour post infection (n = 5). *OAS1*, *IRF7*, *CCL2*, *CXCL10*, and *MSMO1* mRNA expression was analyzed by RT-qPCR and normalized to *GUSB* expression. All data are means ± SEM and were statistically analyzed using two-way repeated measures ANOVA with Holm-Šídák’s multiple comparisons test (*p < 0.05, **p < 0.01, ***p < 0.001; #*p* < 0.05, ##*p* < 0.01, ###*p* < 0.001 (compared to to FBS high/DMSO **(B)**, FBS low/DMSO **(C)**, or FBS low/ctrl **(D)**, respectively)).

As altering ER-to-Golgi dynamics with fatostatin or cholesterol import/export processes via LXR activation both impacted hypoxic ISG induction, we aimed to gain further insights into the potential relevance of subcellular cholesterol dynamics. Since cellular cholesterol homeostasis relies on a tightly regulated interplay between cholesterol uptake, *de novo* synthesis, transport between different compartments, and eventually export (**Figure 5A**), we next addressed ISG induction under conditions when extracellular cholesterol resources are limited. Therefore, we reduced the availability of exogenous cholesterol by lowering the amount of fetal bovine serum (FBS) in the medium. Reduced exogenous cholesterol availability enhanced *OAS1* and *IRF7* expression under normoxia and hypoxia to a similar extend (**Figure 5B**). In contrast, low FBS exclusively enhanced the hypoxic expression of the well-characterized chemokine ISGs *CC motif chemokine ligand 2* (*CCL2*) and *CXC motif chemokine ligand 10* (*CXCL10*), which were previously shown to be induced upon cholesterol disturbances (12). To assess if the ISG-inducing effects of low FBS might indeed be due to decreased uptake of cholesterol, we used the Niemann-Pick C1 protein (NPC1) inhibitor U18666A, which prevents redistribution of LDL-derived cholesterol from lysosomes to cellular organelles such as ER and mitochondria, but also to the plasma membrane (**Figure 5A**). Strikingly, while hypoxic *OAS1* and *IRF7* induction remained unaltered by NPC1 inhibition at high FBS, their serum depletion-dependent increase under hypoxia was prevented (**Figure 5B**). In contrast, *CXCL10* induction by both FBS reduction and/or hypoxia remained largely unaffected, and *CCL2* even increased upon NPC1 inhibition under both normoxia and hypoxia, which was further enhanced when combined with FBS depletion. The differential responses of the ISGs to low serum and/or NPC1 inhibition point towards a complex, cholesterol-associated regulatory network, specific for each ISG. Therefore, we next tested if supplementation of THP-1 cells with methyl-β-cyclodextrin (MβCD)-complexed cholesterol under low serum conditions might affect hypoxic ISG expression patterns. While cholesterol supplementation did not affect *OAS1* and *IRF7* expression at all, it enhanced *CCL2* and *CXCL10* expression predominantly under normoxia (**Figure 5C**). Consequently, the hypoxic induction of chemokine ISGs in serum reduced conditions appeared to be attenuated by cholesterol addition. Not surprisingly, cholesterol supplementation massively reduced both normoxic and hypoxic *MSMO1* expression. Moreover, forced cholesterol loading of THP-1 cells with MβCD-cholesterol under low serum conditions overruled the changes elicited by the NPC1 inhibitor for all ISGs as well as for *MSMO1* (**Figure 5C**). These findings underscore the notion that the impact of intracellular cholesterol dynamics on the expression of ISGs in the context of hypoxia is extremely versatile.

Functionally, interferon-associated immune responses are of special interest when considering virus infections. The β-coronavirus SARS-CoV-2 was first detected in 2019 and described to be the causative agent of a novel lung disease named COVID-19, in which severe clinical manifestations are caused by dysregulated host immune responses (39–41). As COVID-19 is a respiratory disease, which, in severe cases leads to hypoxemia, i.e., low blood oxygen levels, we wondered if hypoxia might influence SARS-CoV-2 infections. Therefore, we incubated THP-1 cells for 24 h under normoxia or hypoxia before adding infectious SARS-CoV-2 (strain FFM1) (18) for 1 h under low serum conditions. Due to technical considerations, all infections had to be carried out under normoxia. Of note, primary monocytes were previously shown to express only low levels of the main SARS-CoV-2 receptor angiotensin-converting enzyme 2 (ACE2) and its associated transmembrane serine protease 2 (TMPRSS2) (42). Despite the fact that THP-1 monocytes only minimally express ACE2, they were substantially infected with SARS-CoV-2 as indicated by the expression of viral *M gene* (C_t_ = 24.10 ± 0.96), yet hypoxic priming only slightly increased virus abundance (**Supplementary Figure 5**). The infection of THP-1 cells was not productive, though, as no active replication of the virus (subgenomic RNA4 encoding E gene) was observed. In line with previous reports suggesting mild interferon responses to SARS-CoV-2 infections (43), *OAS1* and *IRF7* were only slightly elevated in THP-1 cells after infection with SARS-CoV-2 under normoxia and hypoxia. Remarkably, *CCL2* and *CXCL10*, both of which are increased in patients developing a systemic inflammatory response syndrome following SARS-CoV-2 infections (44), showed a strong hypoxic induction upon subsequent infection with SARS-CoV-2 (**Figure 5D**).

Taken together, our data show that cholesterol homeostasis impinges on diverse mechanisms regulating various sub-groups of ISGs under conditions of low oxygen tensions. Of note, hypoxic elevation of chemokine ISGs, which were massively enhanced upon concomitant SARS-CoV-2 infection, appeared to be extremely sensitive to extracellular cholesterol availability and the distribution thereof.

### 3.4 TLR4 signaling contributes to hypoxic ISG induction

**Figure 6:**
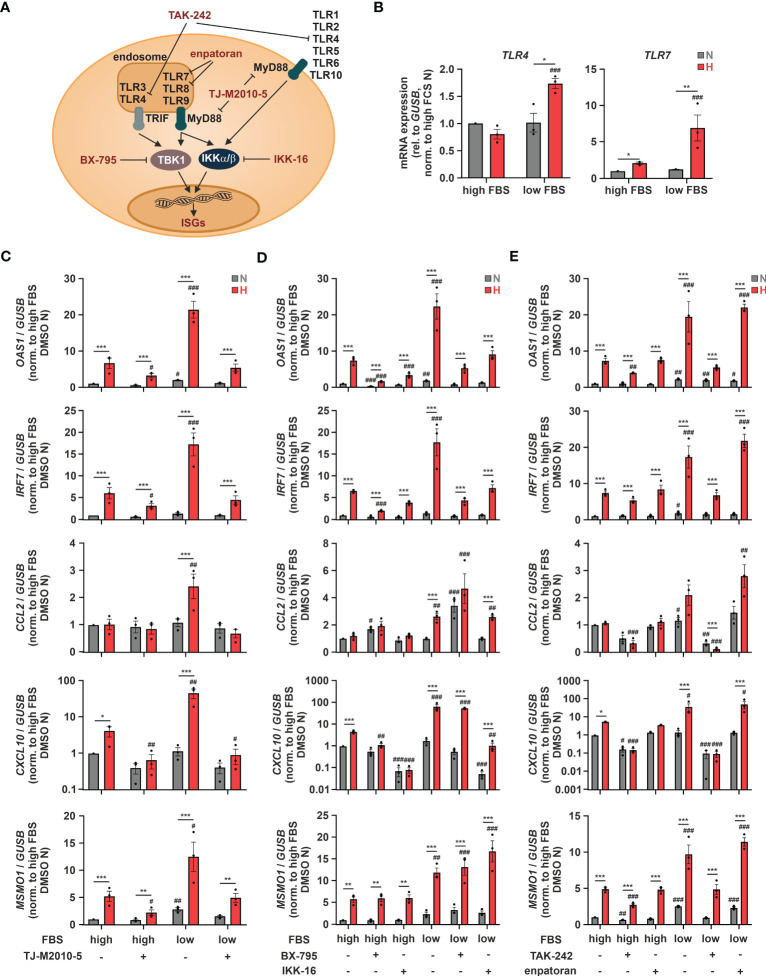
TLR4 signaling contributes to hypoxic ISG induction. **(A)** Overview of toll-like receptors (TLRs), adaptor proteins, and kinases regulating ISG induction. Relevant inhibitors are highlighted in red. **(B)** THP-1 cells were incubated under N (grey) or H (red) for 24 h in medium containing high or low levels of FBS (n = 3). *TLR4* and *TLR7* mRNA expression was analyzed by RT-qPCR and normalized to *GUSB* expression. **(C–E)** THP-1 cells were pre-incubated for 1 h with **(C)** 10 µM TJ-M2010-5 (MyD88 inhibitor), **(D)** 0.5 µM BX-795 (TBK1/IKKϵ inhibitor), 0.1 µM IKK-16 (IKKα/β inhibitor), **(E)** 10 µM TAK-242 (TLR4 inhibitor), 0.1 µM enpatoran (TLR7/8 inhibitor), or DMSO in medium containing high or low levels of FBS prior to incubation under N (grey) or H (red) for 24 h (n = 3). *OAS1*, *IRF7*, *CCL2*, *CXCL10*, and *MSMO1* mRNA expression was analyzed by RT-qPCR and normalized to *GUSB* expression. All data are means ± SEM and were statistically analyzed using two-way repeated measures ANOVA with Holm-Šídák’s multiple comparisons test (*p < 0.05, **p < 0.01, ***p < 0.001; #*p* < 0.05, ##*p* < 0.01, ###*p* < 0.001 (compared to to FBS high **(B)** or FBS high/DMSO **(C–E)**, respectively)).

As hypoxia-enhanced chemokine production in response to SARS-CoV-2 infection might be of major relevance with respect to COVID-19-related systemic inflammation, we further characterized the underlying regulatory principles. We next aimed to determine potentially involved pattern recognition receptors (PRRs). To this end, we first used THP-1 cells deficient for mitochondrial antiviral signaling protein (MAVS) (16), which integrates activity of retinoic acid-inducible gene I (RIG-1) and melanoma differentiation-associated protein 5 (MDA5) (45), or for stimulator of interferon response cGAMP interactor (STING) (16), which is activated by cyclic GMP-AMP synthase (cGAS) (46). In line with the complex, ISG-specific regulation, hypoxic *OAS1*, *IRF7*, and *CXCL10* induction under low serum conditions was lower in MAVS-deficient cells than in STING-knockout (KO) or the corresponding wildtype (WT) THP-1 cells, while hypoxic *CCL2* induction remained unaltered (**Supplementary Figure 6**). Since neither the cGAS/STING nor the RIG-1/MDA5/MAVS axis appeared sufficient for the hypoxic chemokine ISG induction, we asked if toll-like receptors (TLRs) might be involved as well, since they have been shown to not only regulate classical pro-inflammatory cytokines, but also ISGs (**Figure 6A**) (47). Of the 10 known TLRs, *TLR1*, *TLR2*, *TLR4*, and *TLR9* were most abundant, *TLR5*, *TLR6*, and *TLR7* were expressed at intermediate levels, whereas *TLR3*, *TLR8*, and *TLR10* appeared not to be expressed at all in THP-1 cells (**Supplementary Figure 7A**). While *TLR2* and *TLR5* expression did not change in response to hypoxia and/or serum deprivation, expression of *TLR1*, *TLR6*, *TLR7*, and *TLR9* was enhanced by hypoxia and further increased upon serum depletion (**Figure 6B**; **Supplementary Figure 7B**), as observed for *OAS1*, *IRF7*, and *CXCL10* (**Figure 5B**). Interestingly, similar to *CCL2* (**Figure 5B**), *TLR4* was only elevated under hypoxia at low FBS concentrations (**Figure 6B**). To test a general involvement of TLRs in the hypoxic ISG induction, we inhibited myeloid differentiation primary response 88 (MyD88), the intracellular signal transduction adapter for most TLRs, using TJ-M2010-5 (10 µM) (**Figure 6A**). MyD88 inhibition reduced hypoxic induction of *OAS1* and *IRF7* more efficiently under low serum conditions and completely abrogated hypoxia-induced chemokine ISG expression (**Figure 6C**). While TLR-mediated activation of MyD88/inhibitors of nuclear factor kappa B (NF-κB) kinase α/β (IKKα/β)/NF-κB signaling is well established to drive pro-inflammatory cytokine expression, TLR-dependent ISG induction commonly relies on the TIR-domain containing adaptor-inducing interferon-β (TRIF)/TANK-binding kinase 1 (TBK1)/IKKϵ/IRF axis (48). To shed further light on the involved signaling cascade, we inhibited TBK1 using BX-795 (0.5 µM) or canonical IKKs using IKK-16 (0.1 µM), both of which known to be critical for TLR-dependent activation of ISGs (49). While both TBK1 and IKK inhibition significantly reduced hypoxic induction of *OAS1* and *IRF7*, *MSMO1* expression was not altered (**Figure 6D**). Moreover, the prominent hypoxic induction of the chemokine ISGs *CCL2* and *CXCL10* under low serum conditions remained largely unaffected by TBK1 inhibition, whereas IKK inhibition appeared to efficiently reduce *CXCL10* induction, yet leaving *CCL2* unaltered. These findings not only suggest that MyD88- rather than TRIF-dependent signaling underlies the hypoxic chemokine ISG induction, but again underscore the complexity of mechanisms contributing to the hypoxic elevation of the different ISGs.

Since TLR7 and 8 are activated by single stranded RNA viruses such as SARS-CoV-2, and TLR4 has recently been proposed to be activated by SARS-CoV-2 as well (50), we next asked, if they might be also involved in the hypoxic ISG induction. To this end, we used the selective TLR4 inhibitor TAK-242 (10 µM) or the TLR7/8 inhibitor enpatoran (0.1 µM). While enpatoran efficiently blocked ISG expression induced by the specific TLR7/8 agonist resiquimod (R848) (**Supplementary Figure 8**), it did not affect hypoxic induction of any of the tested ISGs irrespective of the serum conditions (**Figure 6E**). In contrast, TLR4 inhibition not only prevented the low serum-dependent increase of the hypoxic *OAS1* and *IRF7* induction, it further blocked chemokine ISG expression altogether. As a side note, reduced hypoxic *MSMO1* induction after TLR4 or MyD88 inhibition corroborated the bidirectionality between IFN signaling and cholesterol metabolism (14).

In summary, TLR4-dependent signaling appears of major importance for the cholesterol dynamic-associated, hypoxic induction of ISGs in monocytes. Herein, chemokine ISGs, such as *CCL2* and *CXCL10*, displayed the strongest addiction to intact TLR4/MyD88 signaling. Moreover, owing to the hypoxic upregulation of various TLRs, a general sensitization of TLR signaling under hypoxia might be predicted.

### 3.5 Hypoxic priming increases the production of chemokine ISGs after SARS-CoV-2 infection via TLR4 activation

**Figure 7:**
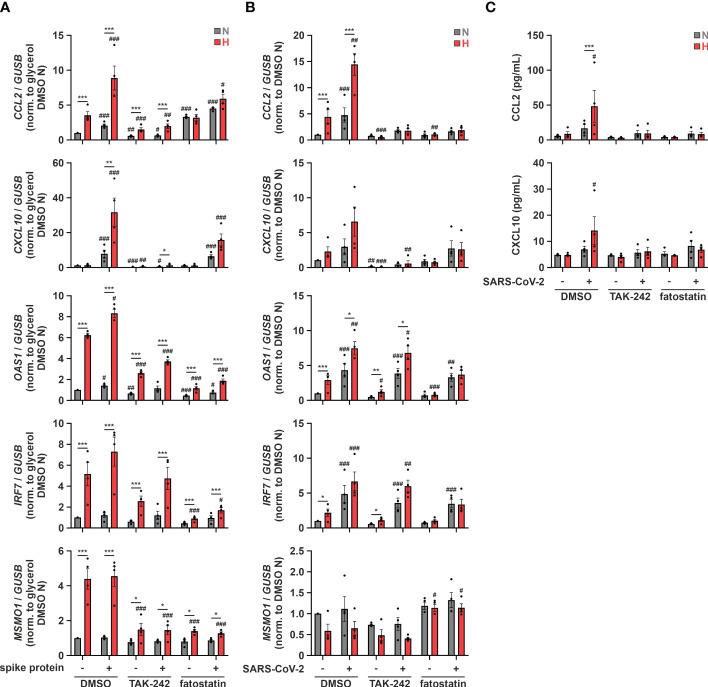
Hypoxic priming increases production of chemokine ISGs after SARS-CoV-2 infection via TLR4 activation. **(A)** THP-1 cells were pre-incubated for 1 h with 10 µM TAK-242, 10 µM fatostatin, or DMSO in medium containing low levels of FBS prior to incubation under N (grey) or H (red) for 32 h 5 µg/mL SARS-CoV-2 spike protein or glycerol were added for the last 8 h (n = 4). *CCL2*, *CXCL10*, *OAS1*, *IRF7*, and *MSMO1* mRNA expression was analyzed by RT-qPCR and normalized to *GUSB* expression. **(B, C)** THP-1 cells were pre-incubated for 1 h with 10 µM TAK-242, 10 µM fatostatin, or DMSO in medium containing low levels of FBS prior to incubation under N (grey) or H (red) for 24 h Subsequently, cells were infected with SARS-CoV-2 (strain FFM1) under N (n = 4). **(B)** RNA was isolated 6 hours post infection. *CCL2*, *CXCL10*, *OAS1*, *IRF7*, and *MSMO1* mRNA expression was analyzed by RT-qPCR and normalized to *GUSB* expression. **(C)** Secreted CCL2 and CXCL10 protein levels were determined by ELISA in supernatants 6 hours post infection. All data are means ± SEM and were statistically analyzed using two-way repeated measures ANOVA with Holm-Šídák’s multiple comparisons test (*p < 0.05, **p < 0.01, ***p < 0.001; #*p* < 0.05, ##*p* < 0.01, ###*p* < 0.001 (compared to to FBS low/DMSO)).

Since TLR4 contributed to hypoxic ISG induction and relevant ISGs increased after SARS-CoV-2 infection in monocytic THP-1 cells, and further taking into account that a direct binding and activation of TLR4 by SARS-CoV-2 spike protein was recently proposed (50), we wondered if SARS-CoV-2 spike protein alone might induce the hypoxic phenotype. Therefore, we pre-incubated THP-1 cells in serum-reduced conditions under either normoxia or hypoxia for 24 h, and continued incubations for additional 8 h in the presence or absence of SARS-CoV-2 spike protein (5 µg/mL). While expression of *OAS1* and *IRF7* only minimally increased in the presence of SARS-CoV-2 spike protein, *CCL2* and *CXCL10*, i.e., chemokine ISGs associated with severe cases of SARS-CoV-2 infections, robustly increased (**Figure 7A**). This became evident already under normoxia and was further enhanced under hypoxia. Inhibition of TLR4 with TAK-242 (10 µM) drastically diminished hypoxia- and SARS-CoV-2 spike protein-induced *CCL2* and *CXCL10* expression and also attenuated hypoxic induction of *OAS1* and *IRF7*. Of note, fatostatin (10 µM) diminished hypoxia-enhanced expression of ISGs and *MSMO1* also in the context of stimulation with SARS-CoV-2 spike protein. Having established that SARS-CoV-2 spike protein induces comparable ISG responses in THP-1 monocytes as the infectious virus and enhances chemokine ISG expression in response to hypoxia, we reached out to determine if TLR4 and cholesterol dynamics are also involved in interferon-associated immune responses in monocytic THP-1 cells upon infection with SARS-CoV-2 in a hypoxic environment. To this end, we primed THP-1 cells for 24 h under hypoxia prior to infecting them with SARS-CoV-2 (FFM1 strain) for 6 h under normoxia. Owing to the reoxygenation, expression of the SREBP2 target *MSMO1* was not elevated in hypoxia-primed THP-1 cells after infection (**Figure 7B**). In contrast to the spike protein, SARS-CoV-2 infection induced *OAS1* and *IRF7* expression already under normoxic conditions, still showing a slight enhancement by hypoxic priming. *CCL2* and *CXCL10* mRNA levels on the other hand were comparable in cells infected with SARS-CoV-2 or treated with spike protein only, displaying a marked increase after hypoxic priming. Interestingly, whereas TLR4 inhibition (TAK-242, 10 µM) did not alter enhanced *OAS1* and *IRF7* expression in response to hypoxic priming and SARS-CoV-2 infection, it completely abolished the expression of chemokine ISGs *CCL2* and *CXCL10* (**Figure 7B**), despite the fact that the infection rate was not affected (**Supplementary Figure 9**). Interfering with intracellular cholesterol dynamics using fatostatin (10 µM) selectively prevented the hypoxia-evoked increase of the ISGs, irrespective of the presence or absence of SARS-CoV-2, without affecting the virus infection rate (**Figure 7B**; **Supplementary Figure 9**). To validate the functional relevance of chemokine ISG expression changes in the context of SARS-CoV-2 infection of monocytic cells under conditions of reduced oxygen availability, we finally determined protein amounts of CCL2 and CXCL10 in the supernatants of THP-1 cells. In line with mRNA expression changes, hypoxia markedly enhanced secretion of CCL2 and CXCL10 upon infection with SARS-CoV-2 (**Figure 7C**). Hypoxic induction again was completely abolished when either TLR4 or SCAP-associated trafficking were inhibited.

Our data suggest that hypoxia increases expression of chemokine ISGs in monocytic THP-1 cells upon infection with SARS-CoV-2 by enhancing spike protein-mediated TLR4 signaling. Severe cases of COVID-19 are characterized by hypoxemia, implying that monocytes regularly encounter hypoxic conditions. Our findings therefore provide a concept of how hypoxia might prime monocytes for TLR4-dependent chemokine ISG production in response to SARS-CoV-2 infection, thus potentially contributing to systemic inflammation.

## 4 Discussion

**Figure 8:**
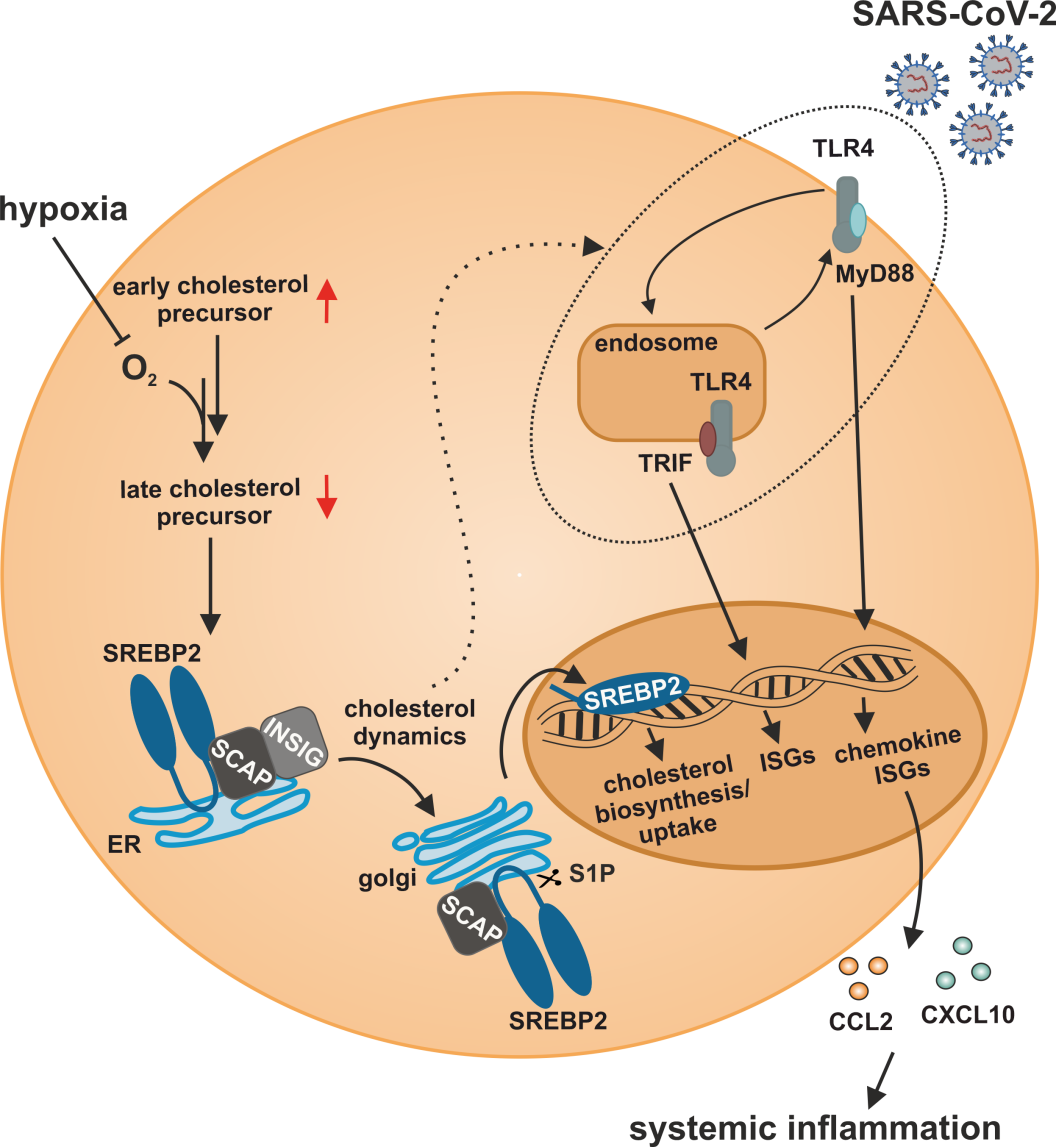
Model of the impact of hypoxia on IFN responses to SARS-CoV-2 infection in monocytes. Hypoxia alters cholesterol biosynthesis flux and subcellular cholesterol dynamics, consequently enhancing TLR4/MyD88-dependent chemokine ISG production in response to SARS-CoV-2 infection.

In this study, we characterized a so far unknown connection between hypoxia-evoked disturbances in cholesterol metabolism and altered IFN responses in monocytes. Cholesterol biosynthesis flux was reduced under hypoxia, resulting in a compensatory SREBP2 activation and consequently enhanced expression of cholesterol biosynthesis enzymes. Also, a broad range of ISGs was induced under hypoxia, but their hypoxic regulation was independent of SREBP2 activity. While a complex regulatory network affected various subgroups of ISGs, intracellular distribution of cholesterol appeared crucial for the hypoxic, TLR4/MyD88-mediated induction of chemokine ISGs. Hypoxia further enhanced chemokine ISG expression in monocytes upon infection with SARS-CoV-2, potentially contributing to systemic inflammatory responses in severe cases of COVID-19 (**Figure 8**).

Our observation that early cholesterol biosynthesis intermediates accumulated, while late intermediates were reduced under hypoxic conditions in THP-1 monocytes, corroborates previous findings of an altered sterol composition under hypoxia (51–53). Moreover, massive and rapid accumulation of lanosterol and 24,25-dihydrolanosterol agrees with previous findings that squalene epoxidase remains active under low oxygen tensions for an extended period of time, thus allowing cholesterol biosynthetic flux to reach lanosterol and 24,25-dihydrolanosterol (51, 54). In contrast to earlier reports claiming that SREBP2 is not activated under hypoxia (55–57), we found a strong feedback activation of SREBP2 despite marginal changes in total cholesterol levels. Supplementation of late cholesterol precursors sufficed to block SREBP2 activity, which suggests that local cholesterol availability (e.g., at the ER) rather than total cholesterol levels are critical in hypoxic monocytes. Differences in sub-cellular cholesterol dynamics or cellular cholesterol requirements might account for the pronounced cell type-specificity of SREBP2 activation in the context of hypoxia. Interestingly, the key enzyme of the cholesterol biosynthesis cascade HMGCR was previously shown to be a direct target of the hypoxia-inducible factor 1 (HIF-1) (58), which might contribute to the enhanced formation of early cholesterol intermediates as well.

Strikingly, expression of IFN response targets closely followed cholesterol biosynthesis changes in hypoxic monocytes, pointing to a potential interplay. Indeed, there is increasing evidence that cholesterol metabolism and IFN responses are tightly interwoven. On the one hand, IFNs and viral infections appear to reduce SREBP2 target expression (14, 15), while on the other hand, cholesterol intermediates were shown to affect ISG expression. Specifically, the early cholesterol intermediate lanosterol repressed IFN signaling in macrophages (59), whereas accumulation of the direct cholesterol precursor 7-dehydrocholesterol or reduced desmosterol levels enhanced ISG expression (13, 60). Furthermore, York et al. (12) provide evidence for an immunometabolic circuit where type I IFN shifts the balance from cholesterol synthesis to uptake, without altering total cholesterol availability. A reduced flux through the cholesterol biosynthesis cascade in turn enhances IFN responses, putatively via lowered cholesterol levels at the ER (12). On a mechanistic side note, SREBP2 was further shown to directly bind to and activate the promotor of various ISGs (38). In contrast, hypoxia-induced ISG expression in monocytes occurred independently of SREBP2, as inhibition of the activating cleavage of SREBP2 by S1P did not attenuate hypoxic ISG induction. Surprisingly though, fatostatin, a commonly used SREBP2 inhibitor, efficiently reduced hypoxia-dependent ISG expression. Fatostatin prevents SREBP2 activation by inhibiting SCAP-coupled ER-to-Golgi transfer. SCAP-associated trafficking was further shown to enhance inflammatory and IFN signaling independent of SREBP2 activation (9, 61). Since cholesterol supplementation did not affect hypoxic *OAS1* and *IRF7* expression, a direct SCAP effect appeared unlikely. Considering SCAP-independent effects of fatostatin, such as a general interference with ER-to-Golgi transport or the inhibition of tubulin polymerization (62, 63), hypoxic ISG induction in monocytes might result from altered intracellular trafficking processes due to changes in subcellular cholesterol distribution instead. Indeed, cholesterol has been shown to influence intracellular trafficking by altering membrane properties or regulating motor proteins (64, 65). As a side note, differentiated, primary macrophages did not show the same phenotype as the monocytic THP-1 cells (data not shown), which might be due to differences in cell culture conditions, likely affecting cellular cholesterol availability and uptake. Yet, further studies are needed to elucidate the exact differences in hypoxic ISG induction between monocytes and macrophages, also with respect to the functional relevance in the context of inflammatory diseases.

Our data suggest that elevated ISG expression under hypoxia demands sensitization of TLR signaling linked to altered intracellular cholesterol dynamics. In fact, associations between cholesterol distribution and TLR trafficking are well-characterized (66, 67) and intracellular trafficking (e.g., between plasma membrane, endosome, or lysosome) has been shown to be critical for activation and termination of TLR signaling as well as recycling (68). Localization is of particular importance in the case of TLR4. TLR4 exclusively activates MyD88-dependent NF-κB signaling when it resides at the plasma membrane, whereas endosomal TLR4 additionally activates TRIF to elicit type I IFN signals (69). In line, accumulation of both TLR4 and cholesterol in endosomal compartments was previously shown to enhance NF-κB as well as IFN signaling upon LPS-treatment in NPC1-deficient cells (70).The observation that TLR4-dependent ISG induction prevailed in hypoxic THP-1 monocytes agrees with the previously described TLR4-dependent upregulation of IFN responses in microglia during ischemia/reperfusion (71). The strict dependency of the hypoxic chemokine ISG induction on MyD88 further corroborates a recent report showing that the direct interaction of cholesterol with MyD88 contributes to signaling amplification of the latter (72). Strikingly, hypoxic induction of chemokine ISGs in monocytes appeared particularly sensitive to changes in intracellular cholesterol trafficking as they showed a more pronounced increase in the hypoxic induction compared to the other ISGs when either extracellular cholesterol availability was reduced or the proper distribution of extracellularly supplied cholesterol was attenuated by inhibition of NPC1. Considering that NPC1 inhibition interferes with cholesterol distribution not only to the ER but also to mitochondria, it can be speculated that changes in mitochondrial integrity, due to altered sterol shuttling to the mitochondria, might contribute to hypoxic ISG induction as well. In line, it is well established that mitochondrial DNA in the cytosol elicits interferon responses (73). Nevertheless, this mechanism was proposed to rely on intact cGAS/STING signaling, which appeared to be not required for the hypoxic ISG induction in THP-1 cells.

While IFN responses are of specific relevance in the context of viral infections (74), SARS-CoV-2 infections were initially deemed to elicit only low levels of type I and III IFNs (41). Interestingly, while we observed a moderate induction of *OAS1* and *IRF7*, chemokine ISGs were markedly induced in response to SARS-CoV-2 infection in hypoxic monocytes. As inhibition of TLR4 activation or interference with cholesterol dynamics completely abolished the enhanced IFN response, TLR sensitization under hypoxia emerged as a potential mechanism. While cholesterol dynamics appeared to be crucial for the hypoxic elevation of all ISGs in the context of SARS-CoV-2 infection, TLR4 was specifically relevant for enhanced chemokine ISGs. Considering the complex regulation of IFN responses via various PRRs, the observation that MAVS also contribute to the hypoxic induction of some ISGs further supports the notion that the hypoxic ISG response integrates numerous receptor-dependent but also -independent signals. Monocytes and monocyte-derived macrophages were previously proposed to resist infection with SARS-CoV-2 due to only minimal expression of the main SARS-CoV-2 receptor ACE2 and its associated serine protease TMPRSS2 (42). Here, we observed infection of THP-1 monocytes with SARS-CoV-2, which corroborates recent findings that monocytes, despite the lack of intrinsic ACE2 expression, can be infected by SARS-CoV-2 (75). In line with previous observations that monocytes/macrophages show no productive infection with SARS-CoV-2 (76), we also did not detect active replication (subgenomic RNA4 encoding E gene). Still, increased production of ISGs and IFN-mediated inflammatory responses after SARS-CoV-2 infection of monocyte-derived cells were previously described (76, 77) and the relevance of monocytes/macrophages with respect to the clinical outcome of COVID-19 is widely accepted (78, 79). Our data suggest that hypoxia-enhanced chemokine ISG responses to SARS-CoV-2 are mediated via TLR4, which likely is activated via the spike protein, as recently suggested (50). Nevertheless, while the exact role of IFN signaling for the pathogenesis of COVID-19 is still controversially discussed and likely depends on the stage of the disease (80–82), our finding that hypoxia specifically enhances chemokine ISG expression in the context of SARS-CoV-2 infection suggests that monocytes within a hypoxemic environment might contribute to the progression from a local inflammatory disease to a systemic inflammatory response syndrome (83–85). Cholesterol homeostasis was not only identified to be important for the infection with SARS-CoV-2 (86), but activation of SREBP2 in blood mononuclear cells was put forward as an indicator of disease severity as it correlated with the development of a cytokine storm in severe cases of COVID-19 (85).

Taken together, we identified hypoxia-mediated changes in cholesterol homeostasis to induce interferon responses in monocytes. Our finding that hypoxic monocytes produce elevated chemokine ISG levels upon SARS-CoV-2 infection in a TLR4- and cholesterol-dependent manner might open new therapeutic opportunities to prevent systemic progression of severe COVID-19 cases.

## Data availability statement

The datasets presented in this study can be found in online repositories. The names of the repository/repositories and accession number(s) can be found in the article/**Supplementary Material**.

## Author contributions

RB, DF, TS and BB conceived the study and designed the experiments. RB and SM performed the experiments. VR, GC, SR, RR, MH and MP contributed to the acquisition of data. DL conducted the sterol measurements. RB, KZ and TS analyzed the data. FR, AW and MW supported the BSL3 experiments. RB and TS wrote the original draft. MW, TS and BB acquired funding. All authors contributed to the article and approved the submitted version.

## Supplementary material

Supplementary Table 2 Hypoxia-induced gene expression changes and associated functions.

Supplementary Table 3 Putative hypoxia-induced ISGs identified by Interferome.
