# Supplementary material for: Hypoxia-altered cholesterol homeostasis enhances the expression of interferon-stimulated genes upon SARS-CoV-2 infections in monocytes
Source: Front Immunol. 2023 Jun 12;14:1121864. doi: 10.3389/fimmu.2023.1121864 (PMC10291055; doi:10.3389/fimmu.2023.1121864)
Supplement: Supplementary file 1 [file DataSheet_1.pdf]

Supplementary Material

Hypoxia-altered cholesterol homeostasis enhances the expression of interferon-stimulated genes upon SARS-CoV-2 infections in monocytes

Rebekka Bauer, Sofie Patrizia Meyer, Rebecca Raue, Megan Alexandra Palmer, Vanesa Maria Guerrero Ruiz, Giulia Cardamone, Silvia Rösser, Fabian Roesmann, Alexander Wilhelm, Dieter Lütjohann, Kathi Zarnack, Dominik Christian Fuhrmann, Marek Widera, Tobias Schmid*, Bernhard Brüne

*** Correspondence:** Tobias Schmid: t.schmid@biochem.uni.frankfurt.de

1 Supplementary Figures and Tables

1.1 Supplementary Figures

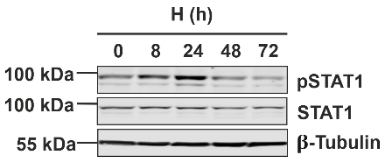

Supplementary Figure 1: Hypoxia-induced STAT1 activation in THP-1 cells. THP-1 cells were incubated under hypoxia (H) for the indicated times. pSTAT1 (Tyr701) and STAT1 protein expression was determined by Western blot analysis. β-tubulin served as loading control. The blot is representative of six independent experiments.

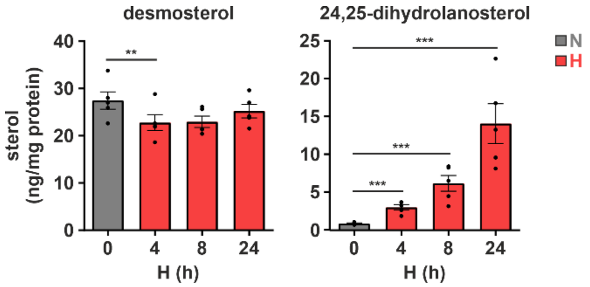

Supplementary Figure 2: Effect of hypoxia on sterol levels. THP-1 cells were incubated under normoxia (N, grey) or H (red) for the indicated times (n = 5). Sterol levels were measured by GC-MS. Data are means ± SEM and were statistically analyzed using one-way repeated measures ANOVA with Holm-Šídák's multiple comparisons test (**$p < 0.01$, ***$p < 0.001$).

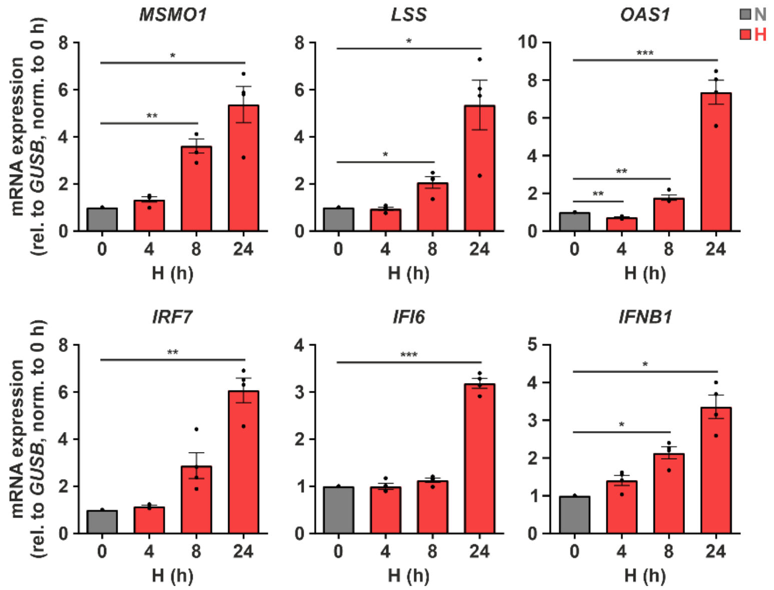

Supplementary Figure 3: Hypoxia induces cholesterol biosynthesis enzymes and interferon-stimulated genes (ISGs). THP-1 cells were incubated under N (grey) or H (red) for the indicated times (n = 4). *MSMO1*, *LSS*, *OAS1*, *IRF7*, *IFI6*, and *IFNB1* mRNA expression was analyzed by RT-qPCR and normalized to *GUSB* expression. Data are means ± SEM and were statistically analyzed using one-way repeated measures ANOVA with Holm-Šídák's multiple comparisons test (*$p < 0.05$, **$p < 0.01$, ***$p < 0.001$).

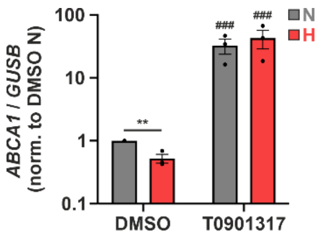

Supplementary Figure 4: Effect of hypoxia and LXR activation on mRNA expression of the cholesterol exporter *ABCA1*. THP-1 cells were pre-incubated for 1 h with 1 µM T0901317 or DMSO prior to incubation under N (grey) or H (red) for 24 h (n = 3). *ABCA1* mRNA expression was analyzed by RT-qPCR and normalized to *GUSB* expression. Data are means ± SEM and were statistically analyzed using two-way repeated measures ANOVA with Holm-Šídák's multiple comparisons test (**$p < 0.01$, ***$p < 0.001$; # compared to DMSO).

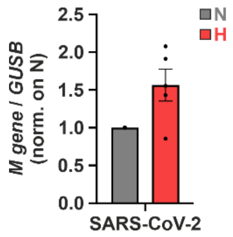

Supplementary Figure 5: Effect of hypoxia on SARS-CoV-2 infection of THP-1 cells. THP-1 cells were incubated for 24 h under N (grey) or H (red) in medium containing low levels of FBS prior to infection with SARS-CoV-2 (strain FFM1) under N. RNA was isolated 1 hour post infection. SARS-CoV-2 *M gene* was quantified by RT-qPCR and normalized to *GUSB* expression. Data are means ± SEM (n = 5) and were statistically analyzed using two-tailed paired t-test.

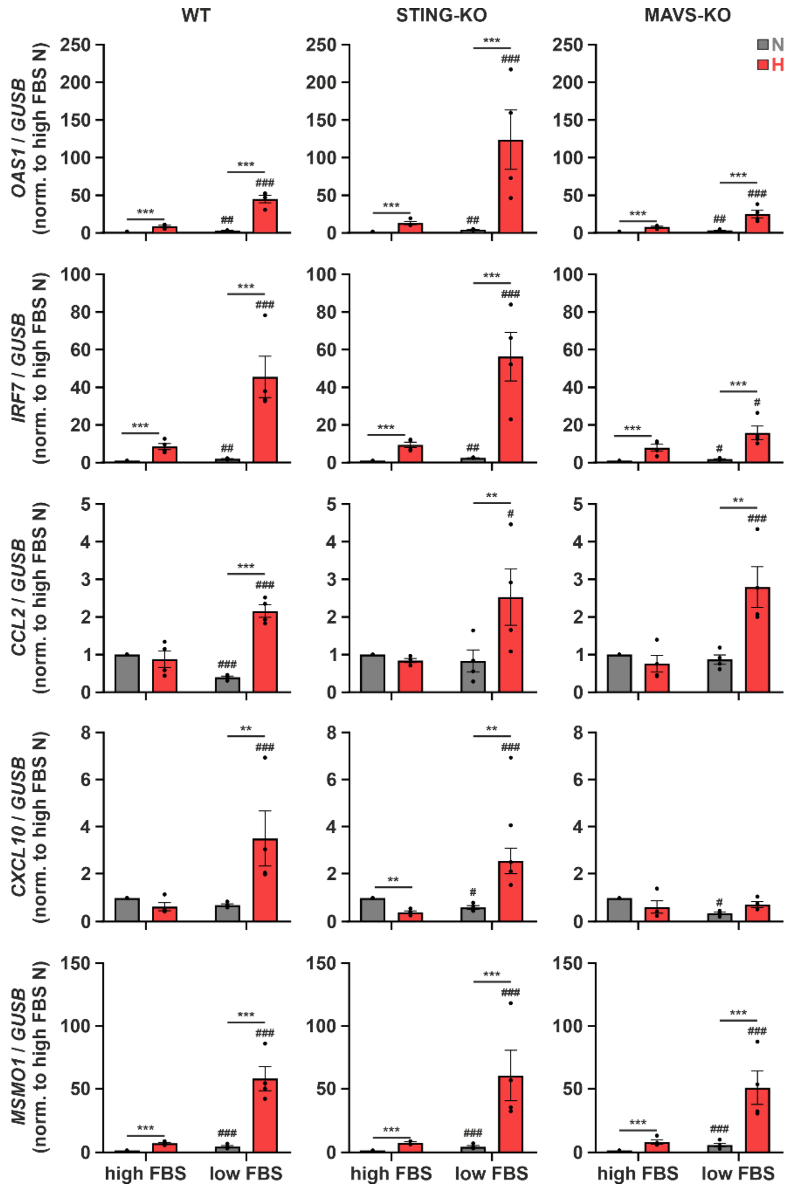

Supplementary Figure 6: Effect of STING or MAVS deficiency on hypoxic ISG/MSMO1 induction. WT, STING-, or MAVS-knockout (KO) THP-1 cells were incubated under N (grey) or H (red) for 24 h in medium containing high or low levels of FBS (n = 4). *OAS1*, *IRF7*, *CCL2*, *CXCL10*, and *MSMO1* mRNA expression was analyzed by RT-qPCR and normalized to *GUSB* expression. Data are means ± SEM and were statistically analyzed using two-way repeated measures ANOVA with Holm-Šídák's multiple comparisons test (**p* < 0.05, ***p* < 0.01, ****p* < 0.001; # compared to FBS high).

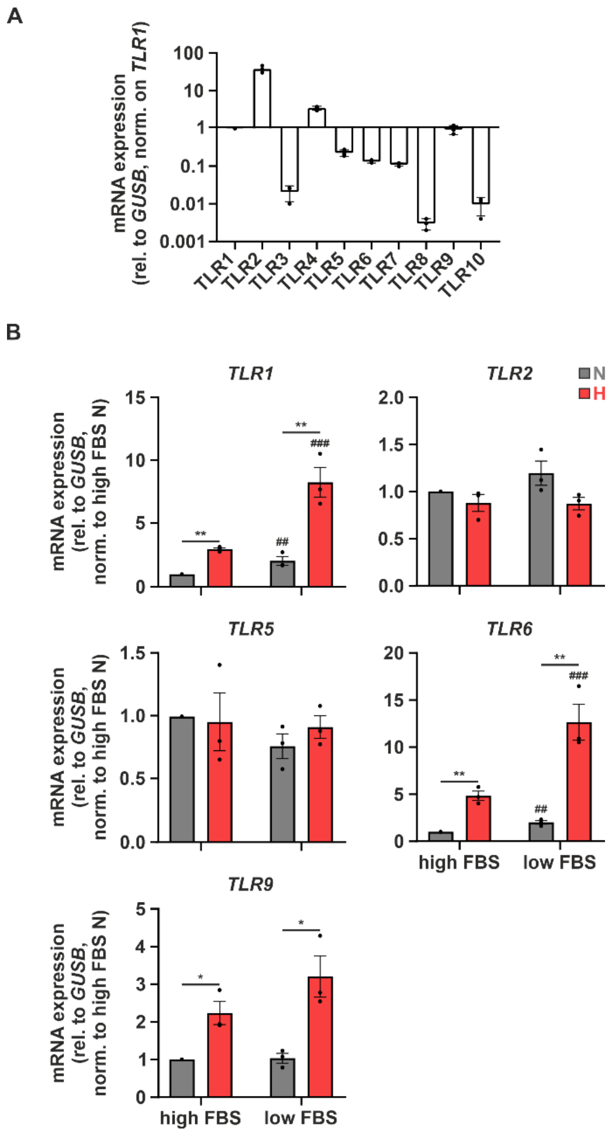

Supplementary Figure 7: TLR expression in THP-1 cells. (A) THP-1 cells were incubated under N for 24 h in medium containing high levels of FBS (n = 3). **(B)** THP-1 cells were incubated under N (grey) or H (red) for 24 h in medium containing high or low levels of FBS (n = 3). mRNA expression of *TLRs 1 - 10* was analyzed by RT-qPCR and normalized to *GUSB* expression. Data are means ± SEM and were statistically analyzed using two-way repeated measures ANOVA with Holm-Šídák's multiple comparisons test (**p* < 0.05, ***p* < 0.01, ****p* < 0.001; $^{\#}$ compared to FBS high).

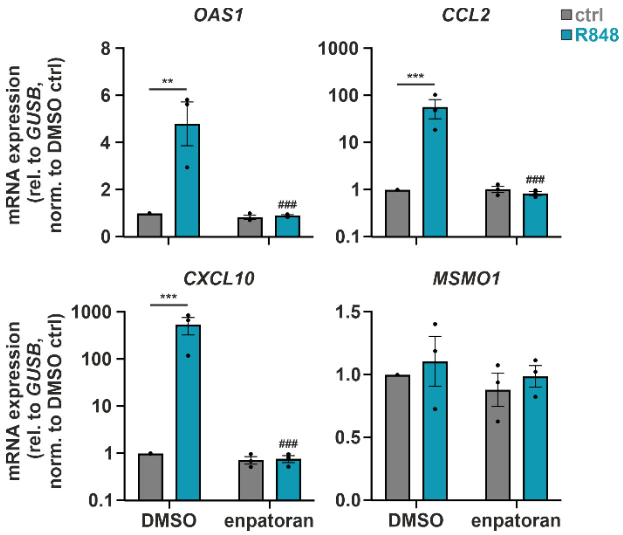

Supplementary Figure 8: Efficiency of TLR7/8 inhibitor enpatoran in THP-1 cells. THP-1 cells were pre-incubated for 1 h with 0.1 µM enpatoran or DMSO prior to incubation with 1 µg/mL resiquimod (R848 (Invivogen, Toulouse, France), blue) or under control conditions (grey) for 24 h (n = 3). *OAS1*, *CCL2*, *CXCL10*, and *MSMO1* mRNA expression was analyzed by RT-qPCR and normalized to *GUSB* expression. Data are means ± SEM and were statistically analyzed using two-way repeated measures ANOVA with Holm-Šídák's multiple comparisons test (***p* < 0.01, ****p* < 0.001; # compared to DMSO).

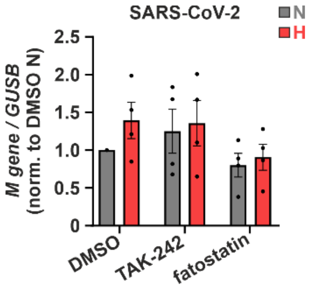

Supplementary Figure 9: Role of TLR4 and cholesterol on SARS-CoV-2 infection of THP-1 cells. THP-1 cells were pre-incubated for 1 h with 10 µM TAK-242, 10 µM fatostatin, or DMSO in medium containing low levels of FBS prior to incubation under N (grey) or H (red) for 24 h. Subsequently, cells were infected with SARS-CoV-2 (strain FFM1) under N (n = 4). RNA was isolated 6 hours post infection. SARS-CoV-2 *M gene* was quantified by RT-qPCR and normalized to *GUSB* expression. Data are means ± SEM and were statistically analyzed using two-way repeated measures ANOVA with Holm-Šídák's multiple comparisons test.

1.2 Supplementary Tables

Supplementary Table S1: Primers used in this study.

primer	forward	reverse
ABCA1	GCTTTCAATCATCCCCTGAA	TGACAGGCTTCACTCCACTG
CCL2	GTCCCAAAGAAGCTGTGATCTTCA	TGGGTTGTGGAGTGAGTGTT
CXCL10	GTGGCATTCAAGGAGTACCTC	TGATGGCCTTCGATTCTGGATT
GUSB	CATTCCTATGCCATCGTGTGGG	GGGGGTGAGTGTGTTGTTGAT
IFI6	CCATCTATCAGCAGGCTCCG	CACCCCACTGCAAGTGAAGA
IFNB1	GCGACACTGTTCGTGTTGTC	GCCTCCCATTCAATTGCCAC
LSS	GCACTGGACGGGTGATTATGG	CAGCCGGATACAGAGAAGAGA
MSMO1	AGCATCCTTGGCTGTGGAAT	CCCATGTCTCTGGCTTATCCTTT
OAS1	ACAGGAAACTTGGGTGGTGG	CTGGGATCGTCGGTCTCATC
SARS-CoV-2 M gene	TGTGACATCAAGGACCTGCC	CTGAGTCACCTGCTACACGC
TLR1	GCACCCCTACAAAAGGAATCTG	TAGGAACGTGGATGAGACCG
TLR2	ATCCTCCAATCAGGCTTCTCT	GGACAGGTCAAGGCTTTTTACA
TLR3	GCTAGCAGTCATCCAACAGAATC	TGGCGGCTGGTAATCTTCTG
TLR4	CAACCTCCCCTTCTCAACCAA	AATTGTCTGGATTTCACACCTGGA
TLR5	ACAGTCACCAAACCAGGGATG	TTCCTGTCTCCAGGTTCGGA
TLR6	TCTTCCTCCTGAAAGCAGAAGT	TTCCGTCGGAGAACTGGATTC
TLR7	GGCCCATCTCAAGCTGATCT	GTGTCCACATTGGAAACACCATT
TLR8	TGGGAAAGGAGACTAAAAAGGAAA	TCTTCGGCGCATAACTCACA
TLR9	AGCATCCTTCCCTGTAGCTG	TGCGGCAGAAACCCATGC
TLR10	TGCAAGCCGTGGGAATTCAG	ATGTCTTCAGGTTGGTGGCA
